# Can the triglyceride–glucose index identify prediabetes in children and adolescents with obesity? a cross-sectional study

**DOI:** 10.3389/fendo.2025.1657912

**Published:** 2025-09-25

**Authors:** Hongxia Liu, Luxin Wang, Xinyu Zhu, Yan Wang, Bo Zhang, Caixia Ma, Li Gao, Jinming Yu

**Affiliations:** ^1^ Department of Pediatrics, Henan Provincial People’s Hospital, People’s Hospital of Zhengzhou University, People’s Hospital of Henan University, Zhengzhou, Henan, China; ^2^ School of Public Health, Fudan University, Shanghai, China

**Keywords:** children with obesity, prediabetes mellitus, TyG index, diabetes screening, type 2 diabetes mellitus

## Abstract

**Purpose:**

This study aims to evaluate the triglyceride–glucose (TyG) index as a biomarker for identifying prediabetes mellitus (PDM) in children and adolescents with obesity and to compare its predictive ability with traditional indicators.

**Methods:**

A cross-sectional study was conducted with 213 children and adolescents with obesity, aged 8 to 18 years. Key inclusion criteria included obesity, defined as a BMI above the 95th percentile. Diagnostic criteria were based on fasting plasma glucose (FPG) and the oral glucose tolerance test (OGTT). Correlation analysis, least absolute shrinkage and selection operator (LASSO) logistic regression, logistic regression, and ROC curve analysis were performed to assess predictive performance.

**Results:**

The TyG index showed significant positive correlations with triglyceride (TG; *r* = 0.751), hemoglobin A1c (HbA1c; *r* = 0.422), 2hPG (*r* = 0.387), and FPG (*r* = 0.385) (all *p* < 0.001). LASSO regression identified TyG, HbA1c, and homeostasis model assessment of insulin resistance (HOMA-IR) as key predictors. Multivariate logistic regression demonstrated that the TyG index was an independent risk factor for PDM (OR = 13.287; 95% confidence interval [CI]: 5.357–32.956; *p* < 0.001). ROC analysis revealed an AUC of 0.776 (95% CI: 0.715–0.837) for the TyG index, with an optimal cutoff of 8.895, yielding a sensitivity of 55.1% and specificity of 85.2%, outperforming TG, FPG, and HOMA-IR.

**Conclusion:**

The TyG index is a reliable and practical marker for identifying prediabetes in children and adolescents with obesity. Its predictive performance exceeds that of several conventional indicators, providing clinical value for early screening and intervention.

## Introduction

The triglyceride–glucose (TyG) index is derived from triglyceride (TG) and fasting plasma glucose (FPG). It has proven to be a convenient, economical, and relatively simple marker for estimating insulin resistance (IR) and related comorbidities. The TyG index is widely used in clinical settings (1–3). Several cross-sectional studies have demonstrated a negative correlation between the TyG index and markers of pancreatic β-cell function, both early and late. This correlation has been observed not only in individuals with normal glucose tolerance (NGT) but also in those with impaired glucose tolerance (IGT) and type 2 diabetes mellitus (T2DM) (4–7). Moreover, longitudinal cohort studies have shown that higher TyG index values are associated with an increased risk of progression to prediabetes. These studies also found a decreased likelihood of returning to normoglycemia (8–10). In conclusion, these results suggest that the TyG index may become a valuable tool for identifying individuals at high risk for prediabetes and T2DM. It could significantly impact early screening, intervention, and prevention efforts.

However, these studies focused primarily on adults and individuals without obesity rather than on children and adolescents. It should be noted that there has been a significant increase in the overweight/obesity rate among children and adolescents, accompanied by a rapid rise in the incidence of prediabetes and T2DM in the same age group (11). Globally, the overall prevalence of prediabetes in adolescents stands at 8.84%. The prevalence rates of impaired fasting glucose (IFG), IGT, and elevated hemoglobin A1c (HbA1c) are 6.93%, 2.59%, and 9.88%, respectively, with notably higher rates observed among adolescents with obesity (12). In the USA, the overall prevalence of prediabetes (defined by IFG, IGT, or elevated HbA1c) among adolescents aged 12–18 years is 18.0% (95% confidence interval [CI]: 16.0%–20.1%). Notably, the prevalence is significantly higher in adolescents with obesity (25.7%; 95% CI: 20.0%–32.4%) compared with their peers with normal weight (16.4%; 95% CI: 14.3%–18.7%) (13). Furthermore, the phenotype of prediabetes is more severe in children and adolescents than in adults. This is demonstrated by a longer progression period from prediabetes to T2DM in adults, which typically takes 5–10 years. In contrast, this progression occurs within only 2–3 years in children and adolescents (14, 15). This difference may be linked to their higher levels of IR and β-cell dysfunction (16). Consequently, early identification and management of prediabetes in these high-risk populations can potentially mitigate the risk of developing T2DM and its associated complications later, particularly in children and adolescents.

Although guidelines advocate for targeted screening for prediabetes in children and adolescents exhibiting high-risk factors, such as obesity or a familial predisposition to T2DM, the optimal screening test or strategy is still a topic of debate (17–19). For instance, IGT frequently goes undetected by FPG alone in children with prediabetes. Furthermore, despite the oral glucose tolerance test (OGTT) being widely acknowledged as the gold standard, its use in clinical screening is limited by its time-consuming, expensive, and poorly reproducible nature. Additionally, the sensitivity of hemoglobin A1c (HbA1c) in prediabetes detection is influenced by factors such as race, age, disease condition, and the methodology employed for its detection (20). Numerous adult studies have found that the TyG index is a better predictor than other traditional measures such as FPG, TG, and HbA1c (21, 22). Multiple cross-sectional studies have indicated a significant positive correlation between the TyG index and insulin resistance, as well as other cardiometabolic risk factors, in children and adolescents (23, 24). A study by Mohd Nor et al. (25) conducted in adolescents with obesity found that the TyG index was significantly negatively correlated with insulin sensitivity measured by the hyperinsulinemic–euglycemic clamp (*r* = − 0.419, *p* < 0.0001). Moreover, the TyG index showed a progressively increasing trend across glucose tolerance categories—normal glucose tolerance, prediabetes, and T2DM—with values of 8.3 ± 0.5, 8.6 ± 0.5, and 8.9 ± 0.6, respectively (*p* < 0.0001). The study also identified an optimal TyG index cutoff value of 8.52 for diagnosing insulin resistance, with a sensitivity of 69.1% and specificity of 71.7%. Additionally, the TyG index demonstrates superior performance in predicting metabolic syndrome (MetS), with cutoff values ranging from 8.33 to 8.47, yielding sensitivities between 75% and 90% and specificities between 67% and 78% (26). Although the TyG index shows promising application potential in pediatric populations, whether these findings are applicable to children and adolescents with prediabetes remains to be established. The standardization of its cutoff values also remains a challenge (27). Therefore, this study aimed to investigate whether the TyG index is associated with prediabetes in Chinese children and adolescents with obesity, and to compare the roles of FPG, TG, HbA1c, and the TyG index in predicting the incidence of prediabetes. To the best of our knowledge, this is the first study to compare the roles of the TyG index, FPG, TG, and HbA1c in predicting prediabetes among Chinese children and adolescents with obesity.

## Materials and method

### Study population

**Figure 1:**
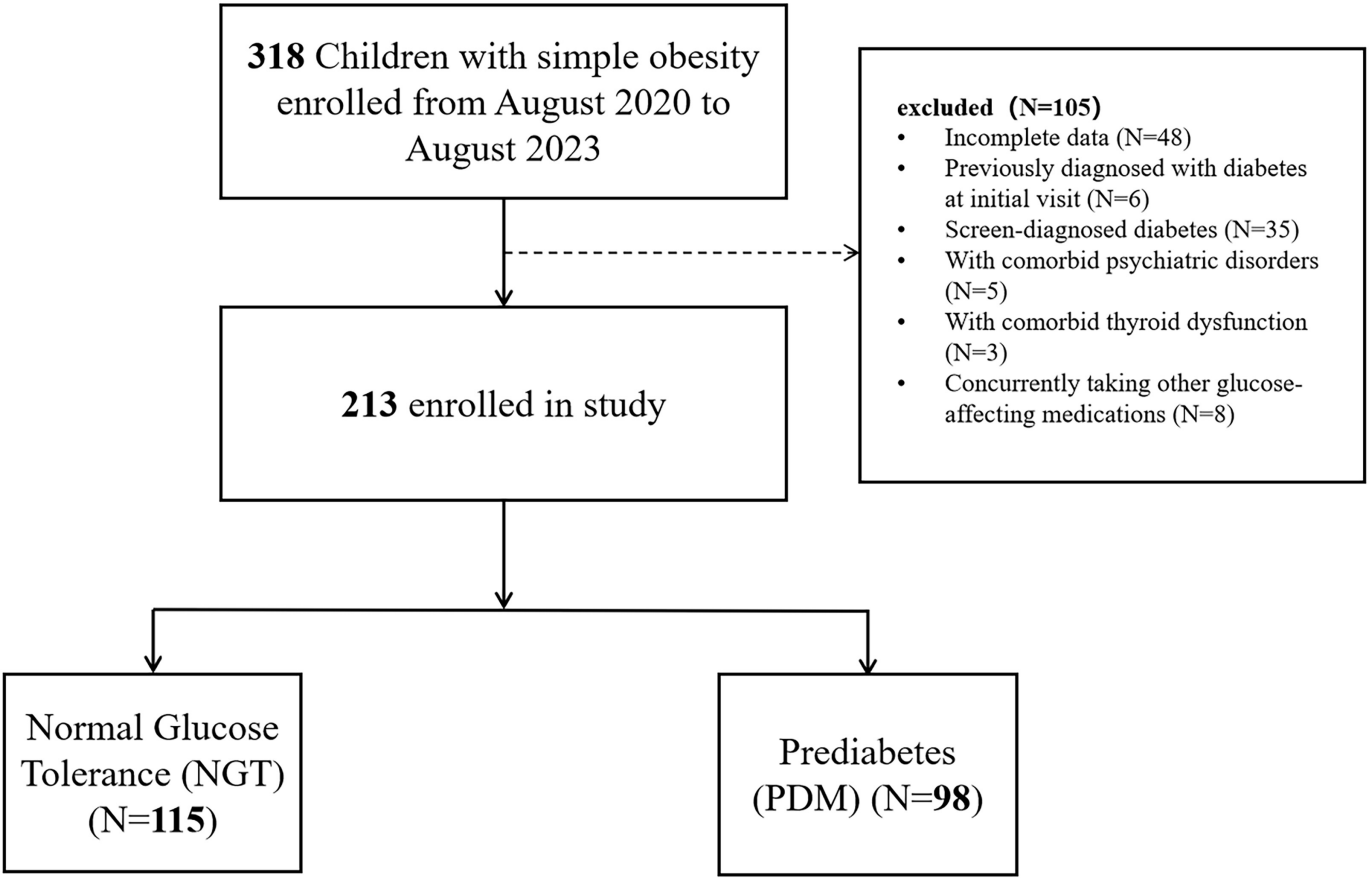
Flowchart of patient selection. (1) NGT: FPG < 6.1 mmol/L and 2-h plasma glucose (2hPG) after glucose load < 7.8 mmol/L. (2) Isolated impaired fasting glucose (iIFG): 6.1 mmol/L ≤ FPG < 7.0 mmol/L and 2hPG < 7.8 mmol/L. (3) Isolated IGT (iIGT): FPG < 6.1 mmol/L and 7.8 mmol/L ≤ 2hPG < 11.1 mmol/L. (4) Combined iIGT and iIGT (iIFG + iIGT): 6.1 mmol/L ≤ FPG < 7.0 mmol/L and 7.8 mmol/L ≤ 2hPG < 11.1 mmol/L. (5) Diabetes mellitus (DM): FPG ≥ 7.0 mmol/L or 2hPG ≥ 11.1 mmol/L.

We collected cross-sectional clinical, anthropometric, and demographic data of 213 individuals with obesity aged between 8 and 18 years who were hospitalized at Henan Provincial People’s Hospital from August 2020 to August 2023 (**Figure 1**). Obesity was defined as having a body mass index (BMI) at or above the 95th percentile for children and adolescents of the same gender and age, with BMI calculated as weight in kilograms divided by height in square meters. Prediabetes and diabetes were diagnosed according to the World Health Organization (WHO) criteria published in 1999 (28).

Participants with isolated impaired fasting glucose (iIFG), isolated IGT (iIGT), or combined iIGT and iIGT (iIFG + iIGT) were classified as having prediabetes. This study was conducted in accordance with the standards of the Declaration of Helsinki on medical research, and all procedures involving human participants were approved by the Ethics Committee of the Henan Provincial People’s Hospital. After the parental informed consent was obtained, participants were excluded if they met any of the following criteria: (1) incomplete data, such as missing key data required for analysis (e.g., fasting plasma glucose, 2-h plasma glucose, or triglyceride measurements); (2) secondary or genetic obesity, including obesity due to endocrine disorders (e.g., hypothyroidism, Cushing’s syndrome) or monogenic obesity syndromes; (3) history of diabetes or significant weight change, defined as prior diagnosis of type 1 or 2 diabetes or unintentional weight loss exceeding 5% of body weight within the past 6 months; (4) other chronic diseases, including major cardiovascular, hepatic, or renal disease, or treatment with medications known to significantly affect glucose or lipid metabolism; (5) severe physical or psychiatric disorders, such as malignancy, severe respiratory or cardiovascular disease, schizophrenia, or severe mood disorders.

### Anthropometric and clinical assessment

An anthropometric standard clinical examination was conducted for each child. All measurements were performed in the morning, with children fasting for at least 10 h and dressed in light underwear and barefoot or wearing socks. Height and weight were measured twice, and the averages were recorded, using a stadiometer with a sensitivity of  ± 0.1 cm and a digital scale with a sensitivity of ± 0·1 kg, respectively. Waist circumference (WC) was measured at the mid-level between the lower rib margin and the iliac crest. The systolic (SBP) and diastolic blood pressures (DBP) were measured twice at 5-min intervals using an Omron sphygmomanometer, and the average was recorded in millimeters of mercury (mmHg).

Fasting blood samples were collected from the children between 08:00 and 09:00 hours for biochemical tests, including fasting blood glucose (FBG), TG, total cholesterol (TC), low-density lipoprotein (LDL), high-density lipoprotein (HDL), HbA1c, liver and kidney function such as alanine aminotransferase (ALT), aspartate aminotransferase (AST), gamma-glutamyl transferase (γ-GGT), alkaline phosphatase (ALP), blood urea nitrogen (BUN), creatinine (Cr), and uric acid (UA), as well as thyroid function markers such as free triiodothyronine (FT3), free thyroxine (FT4), and thyroid-stimulating hormone (TSH). Subsequently, a 3-h OGTT was performed. Each child received an oral glucose load of 1.75 g/kg body weight (maximum dose: 75 g). Venous blood samples were collected at 0 (fasting), 30, 60, 120, and 180 min after glucose ingestion for the measurement of plasma glucose and insulin concentrations.

Based on OGTT results, participants were classified into the NGT group and the prediabetes mellitus (PDM) group according to standard diagnostic criteria.

Routine biochemical measurements of fasting blood samples were performed with an automatic analyzer (Architect C16000, Abbott, Chicago, IL, USA). HbA1C was determined using high-performance liquid chromatography (Lifotronic H9, Lifotronic instrument, Shenzhen, China; reference range: 4.0%–6.1%). Insulin levels were measured with the enzyme-amplified chemiluminescence immunoassay (CLIA) technology (Immulite^®^ 2000 XPi Immunoassay System, Siemens Healthineers, Berlin, Germany).

The TyG index was calculated using the following formula: Ln [TG (mg/dL) × FBG (mg/dL)/2]. To ensure consistency with international standards, if FBG was initially measured in millimoles per liter, the conversion to milligrams per deciliter must be made using the following conversion factor: FBG (mg/dL) = FBG (mmol/L) × 18.018 FBG (mg/dL) = FBG (mmol/L) × 18.018 (29). Insulin resistance was estimated using the homeostasis model assessment of insulin resistance (HOMA-IR) as described by Matthews et al. (30), calculated as FBG (mmol/L) × fasting insulin (µIU/mL)/22.5.

### Statistical analysis

Anthropometrics and clinical assessment. Statistical analysis was performed by SPSS 26.0 software (IBM Co., Armonk, NY, USA) and R (version 4.3.1). Qualitative data were presented as numbers (percentages), and quantitative data were presented as mean (SD) or as median with interquartile range (IQR). The Chi-square test was used for the comparison of categorical variables. The Student’s *t*-test was used to compare normally distributed quantitative data, and the Mann–Whitney *U* test was used to compare nonnormally distributed data.

Pearson correlation analysis was used to analyze the correlation between each metabolic and clinical index and glucose status, with insignificant correlation for *r^2^* values below ± 0.10 and more significant correlation for values above ± 0.40 (31).

Given our moderate sample size (*n* = 213) and the inclusion of multiple traditional metabolic indicators (e.g., TG, FPG, and HbA1c) that may exhibit strong intercorrelations, we employed least absolute shrinkage and selection operator (LASSO) regression to address multicollinearity, mitigate overfitting, and enhance model generalizability. LASSO regression was applied to identify and retain the metabolic and clinical indicators most strongly associated with PDM from the initial set of variables (32).

The receiver-operating characteristic (ROC) curve and logistic analysis were generated to compare the diagnostic performance of the TyG index, FPG, TG, and HbA1c, with sensitivities, specificities, and optimal cutoff points calculated for prediabetes classification. We assessed the overall performance of each index for predicting prediabetes by computing the area under the curve (AUC). The Youden index was used to determine the optimal cutoff value for each indicator. Binary logistic regression modeling was performed to assess the dose-dependent relationship between the TyG index and the risk of PDM as a continuous variable (per 1-unit increment) and when categorized into tertiles (lowest tertile [T1]: ≤ 8.42; intermediate tertile [T2]: 8.43–8.89; highest tertile [T3]: > 8.90).

A *p* < 0.05 was considered statistically significant.

## Results

### Demographic and clinical characteristics of the participants according to glucose tolerance groups

This study included a total of 213 children with obesity. The mean age was 12.86 years ± 2.71 years. There were no significant differences between the two groups in baseline characteristics, including gender, age, BMI, and blood pressure (*p* > 0.05). After evaluation using an OGTT, 115 children (54.0%) were classified as having NGT, while 98 children (46.0%) were classified as having PDM. Among the participants in the PDM group, 12 (12.2%) had impaired fasting hyperglycemia (IFG), 74 children (75.6%) had impaired glucose regulation (IGT), and 12 children (12.2%) had both IFG and IGT. Compared to the NGT group, participants in the PDM group exhibited significantly higher levels of TG, UA, HOMA-IR, TyG index, FBG, 2-h blood glucose (2hBG), and HbA1c (*p* < 0.05), as well as significantly lower levels of high-density lipoprotein cholesterol (HDL-c) (*p* < 0.05). No significant differences were observed between the two groups for other indicators (**Table 1**).

**Table 1:** Demographic and clinical characteristics of the participants according to glucose tolerance groups.

Baseline characteristics	Total (*N* = 213)	NGT (*n* = 115)	Prediabetes (*n* = 98)	*T*/*χ^2^*)/*Z*	*P-*value
Gender (boy/girl) (*n*)	145/68	82/33	63/35	1.199	0.273
Age at the time of visit (years)	12.86 ± 2.71	12.99 ± 2.77	12.70 ± 2.65	0.762	0.447
Family history (*n*)	79 (37.1)	36 (31.3)	43 (43.9)	3.585	0.058
Fatty liver (%)	180 (84.5)	94 (81.7)	86 (87.8)	1.463	0.227
BMI (kg/m (2))	29.59 (27.23, 32.66)	29.59 (27.27, 32.85)	29.58 (26.84, 31.89)	− 0.786	0.432
BMI *Z*-score	2.83 (2.42, 3.27)	2.83 (2.52, 3.35)	2.84 (2.38, 3.11)	− 1.080	0.280
SBP (mmHg)	120.88 ± 13.101	120.76 ± 12.16	121.02 ± 14.19	− 0.146	0.884
DBP (mmHg)	79.10 ± 11.48	79.21 ± 11.41	78.97 ± 11.62	0.151	0.880
Hg (g/L)	134.00 (127.00, 143.00)	134.00 (128.00, 142.00)	134.00 (126.75, 143.00)	− 0.146	0.884
Lipids on admission					
TC (mmol/L)	4.16 (3.64, 4.66)	4.14 (3.53, 4.64)	4.21 (3.66, 4.71)	− 0.722	0.471
TG (mmol/L)	1.35 (1.02, 1.98)	1.22 (0.91, 1.55)	1.78 (1.18, 2.41)	− 5.373	< 0.001^*^
HDL-c (mmol/L)	1.06 (0.94, 1.21)	1.09 (0.96, 1.27)	1.03 (0.92, 1.14)	− 2.526	0.012
LDL-c (mmol/L)	2.48 (2.07, 2.89)	2.47 (2.03, 2.80)	2.49 (2.08, 3.02)	− 1.130	0.260
Biochemical indicators					
ALT (U/L)	28.50 (19.60, 55.50)	27.00 (19.20, 54.30)	30.30 (20.00, 60.35)	− 0.834	0.404
AST (U/L)	23.90 (18.45, 34.90)	22.70 (17.70, 32.70)	25.00 (19.18, 37.23)	− 1.844	0.065
γ-GGT (U/L)	25.00 (17.90, 37.95)	24.60 (16.70, 36.20)	26.15 (19.08, 39.25)	− 1.099	0.272
ALP (U/L)	199.00 (188.50, 270.50)	197.00 (115.00, 262.00)	199.00(121.75, 279.25)	− 0.625	0.532
Serum UA (µmol/L)	403.00 (344.00, 488.00)	384.00 (339.00, 472.00)	410.50 (360.25, 509.50)	− 2.121	0.034
UN (mmol/L)	4.48 ± 1.11	4.48 ± 1.12	4.48 ± 1.12	0.031	0.976
Serum creatinine (µmol/L)	45.00 (39.00, 55.00)	46.00 (41.00, 56.00)	44.00 (37.75, 54.00)	− 1.589	0.112
Thyroid function					
FT3 (pmol/L)	6.23 ± 1.09	6.32 ± 1.10	6.12 ± 1.07	1.344	0.180
FT4 (pmol/L)	16.22 ± 2.94	16.42 ± 3.14	15.98 ± 2.70	1.081	0.281
TSH (µIU/mL)	2.95 (2.01, 4.08)	2.87 (2.01, 4.07)	3.02 (2.01, 4.37)	− 0.162	0.872
Derived indicators					
HOMA-IR	4.54 (3.27, 6.47)	4.33 (3.14, 5.79)	5.00 (3.76, 7.18)	− 2.851	0.004
TyG index	8.69 ± 0.56	8.44 ± 0.46	8.96 ± 0.53	− 7.754	< 0.001^*^
Hyperglycemia on admission					
FBG (mmol/L)	5.20 (4.70, 5.80)	4.93 (4.41, 5.40)	5.64 (5.10, 6.03)	− 6.337	< 0.001^*^
2hBG (mmol/L)	7.30 (6.47, 8.20)	6.70 (6.10, 7.20)	8.35 (7.90, 9.20)	− 11.319	< 0.001^*^
HbA1c (%)	5.40 (5.20, 5.70)	5.30 (5.00, 5.50)	5.65 (5.40, 5.80)	− 6.493	< 0.001*
FINS	20.22 (14.30, 28.27)	19.82 (14.02, 27.24)	20.64 (14.88, 28.73)	− 1.228	0.219

The data are expressed as the mean ± standard deviation, median (interquartile range), or *n* (%).

*2hBG*, 2-h blood glucose; *ALT*, alanine aminotransferase; *ALP*, alkaline phosphatase; *AST*, aspartate aminotransferase; *BMI*, body mass index; *DBP*, diastolic blood pressure; *FBG*, fasting blood glucose; *FINS*, fasting insulin; *FT3*, free triiodothyronine; *FT4*, free thyroxine; *γ-GGT*, gamma-glutamyl transferase; *HbA1c*, hemoglobin A1c; *HDL-c*, high-density lipoprotein cholesterol; *Hg*, hemoglobin; *HOMA-IR*, homeostasis model assessment of insulin resistance; *LDL-c*, low-density lipoprotein cholesterol; *SBP*, systolic blood pressure; *TC*, total cholesterol; *TG*, triglycerides; *TSH*, thyroid-stimulating hormone; *TyG*, triglyceride-glucose; *UA*, uric acid; *UN*, urea nitrogen. Asterisk indicates statistical significance after Bonferroni correction.

### Correlations between the TyG index and clinical characteristics

**Figure 2:**
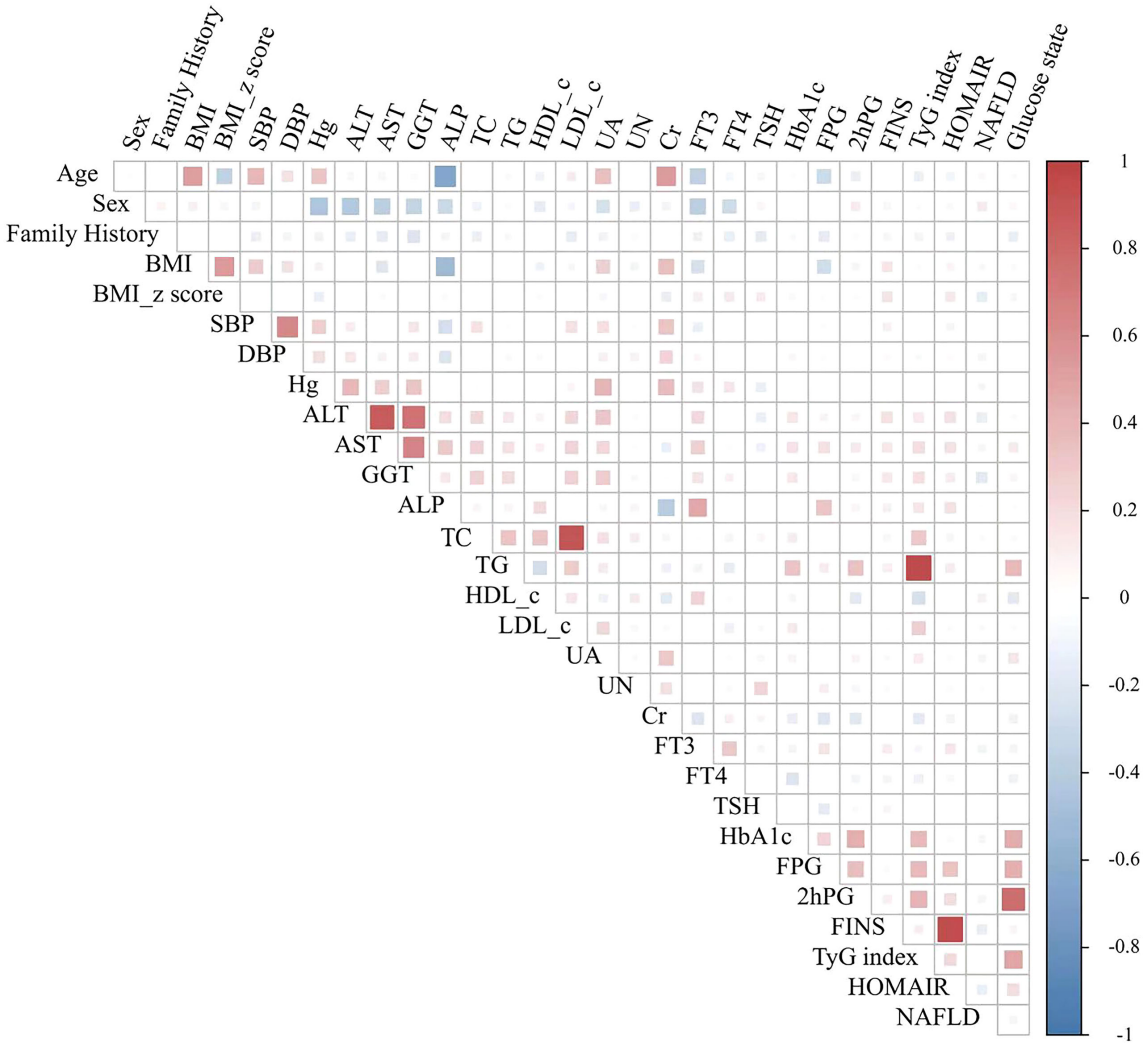
Correlations between TyG index and clinical characteristics. Pearson correlation matrix, with color and size representing the strength and direction of correlation (*r*). SBP, systolic blood pressure; DBP, diastolic blood pressure; Hb, hemoglobin; ALB, albumin; ALT, alanine transaminase; Cr, creatinine; BUN, blood urea nitrogen; TG, triglyceride; TC, total cholesterol; HDL, high-density lipoproteins; LDL, low-density lipoproteins; FT3, free triiodothyronine; FT4, free thyroxine; TSH, thyroid-stimulating hormone; HbA1c, glycated hemoglobin; FBG, fasting blood glucose; TyG, triglyceride glucose index.

The correlations between the TyG index and other clinical characteristics are shown in **Figure 2**. The TyG index was significantly correlated with TG (*r* = 0.751), HbA1c (*r* = 0.422), and TC (*r* = 0.415) (all *p* < 0.001). In addition, the TyG index correlated negatively with HDL-c (*r* = − 0.265, *p* < 0.001). Furthermore, the TyG index was correlated with 2-h plasma glucose (2hPG; *r* = 0.387), FPG (*r* = 0.385), low-density lipoprotein cholesterol (LDL-c; *r* = 0.254), γ-GGT (*r* = 0.224), ALP (*r* = 0.164), ALT (*r* = 0.140), and UA (*r* = 0.136) (all *p* < 0.05), while no significant correlation with other clinical indices was observed (all *p* > 0.05), such as SBP, DBP, BMI, age, gender, fasting insulin (FINS), and HOMA-IR.

### LASSO regression screening for risk factors

**Figure 3:**
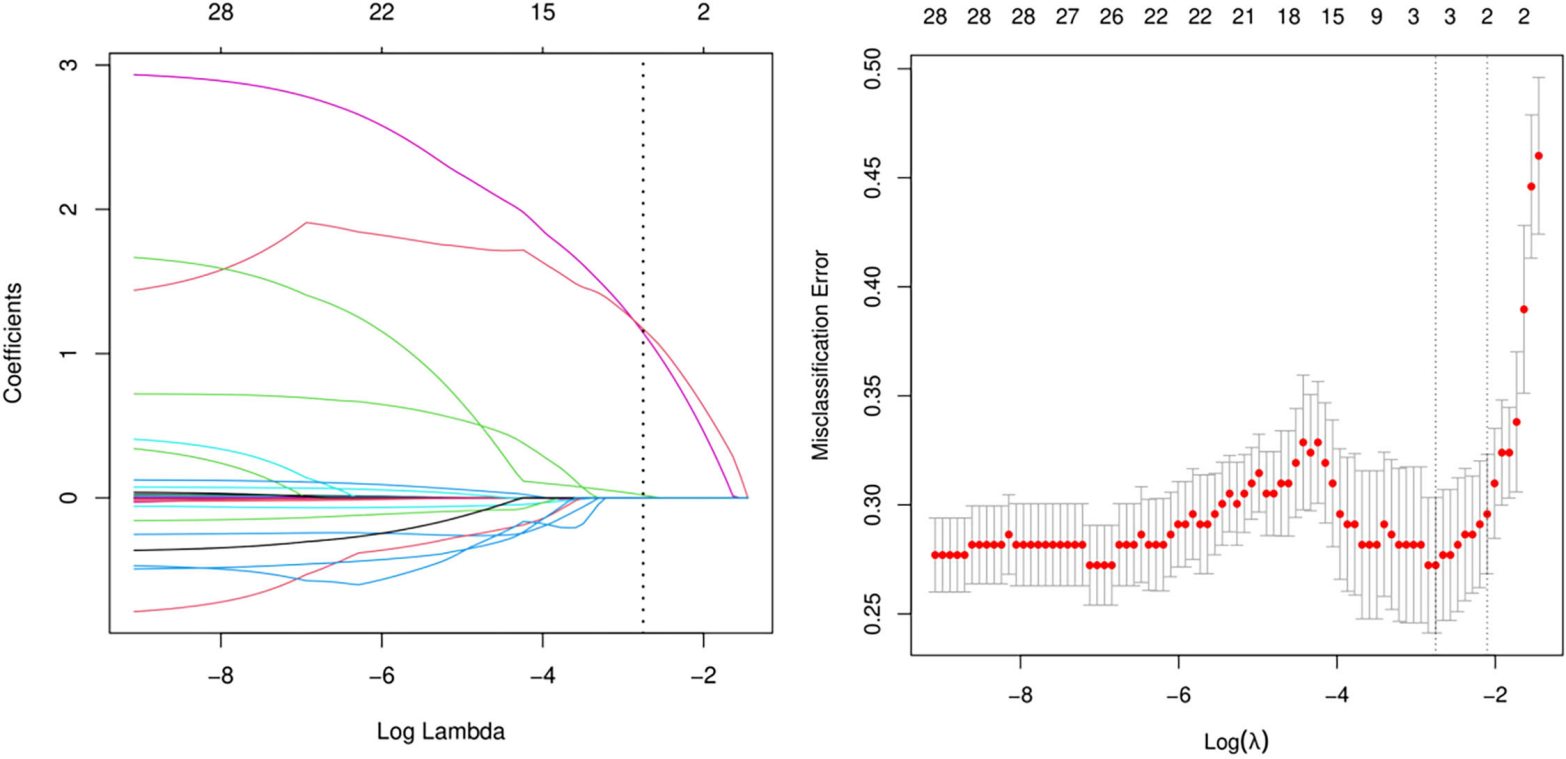
Feature selection using the least absolute shrinkage and selection operator (LASSO) binary logistic regression model. LASSO, least absolute shrinkage and selection operator; SE, standard error. **(A)** Coefficient shrinkage patterns of 28 features across regularization intensities. The coefficient profile illustrates the path of L1-penalized regression coefficients through a geometrically decreasing λ sequence (log-transformed). Three clinically significant features demonstrated nonzero coefficients after optimal shrinkage. **(B)** Parameter selection in the LASSO model using 10-fold cross-validation with the minimum criterion. Misclassification error (CLASS) curves and logarithmic (lambda) curves were plotted. The minimum standard and 1-standard error (1SE) of the minimum standard were used to draw vertical dashed lines at the optimal values. The optimal lambda produced three nonzero coefficients.

The LASSO logistic regression model was applied to identify the most relevant predictors of PDM in children and adolescents with obesity. As shown in **Figures 3A, B**, the optimal penalty parameter (λ) was selected based on the minimum cross-validated misclassification error. Using this criterion, three variables among 28 with nonzero coefficients were retained: the TyG index, HbA1c, and HOMA-IR.

### Logistic regression analysis of independent associations between metabolic markers and PDM

After completing correlation analysis between metabolic markers and prediabetes status and performing variable selection via LASSO regression, we constructed logistic regression models to further evaluate the independent effects of core indicators while controlling for confounding bias. By excluding highly collinear variables (e.g., TC and FINS) while retaining FBG and 2hBG as reference indicators, we present regression results under different adjustment strategies, including an unadjusted model and sequentially adjusted models incorporating demographic characteristics, lipid profiles, liver function, and other covariates.

The results of the binary logistic regression analysis are presented in **Table 2**. In the unadjusted model, the risk of prediabetes was significantly associated with increases in HbA1c (odds ratio [OR]: 14.504, 95% CI: 5.731–36.711, *p* < 0.001), TG (OR: 3.104, 95% CI: 1.971–4.888, *p* < 0.001), TyG index (OR: 10.476, 95% CI: 4.985–21.981, *p* < 0.001), and HOMA-IR (OR: 1.157, 95% CI: 1.048–1.278, *p* = 0.004). After further adjustment for various confounding factors, including gender, age, family history of diabetes, presence of fatty liver, BMI, SBP, DBP, hemoglobin, LDL-c, HDL-c, AST, ALT, γ-GGT, ALP, UA, BUN, Cr, and thyroid function (models 1 and 2), HbA1c, TG, TyG index, and HOMA-IR remained significant risk factors for the development of PDM in children with obesity.

**Table 2:** Logistic regression analysis of the relationship between calculated indices and the risk of prediabetes in children with obesity.

Variable	*B*-value	Standard error of *B*	Wald value	*P*-value	OR	95% CI
2hBG						
Unadjusted	3.005	0.433	48.165	< 0.001	20.188	8.640–47.170
Model 1	3.230	0.478	45.614	< 0.001	25.282	9.902–64.550
Model 2	3.630	0.568	40.842	< 0.001	37.695	12.384–114.739
FBG						
Unadjusted	1.509	0.258	34.227	< 0.001	4.520	2.727–7.493
Model 1	1.602	0.287	31.225	< 0.001	4.962	2.829–8.702
Model 2	1.885	0.343	30.287	< 0.001	6.588	3.366–12.891
HbA1c						
Unadjusted	2.674	0.474	31.863	< 0.001	14.504	5.731–36.711
Model 1	2.799	0.493	32.250	< 0.001	16.432	6.253–43.177
Model 2	2.931	0.548	28.574	< 0.001	18.741	6.399–54.888
TG						
Unadjusted	1.133	0.232	23.893	< 0.001	3.104	1.971–4.888
Model 1	1.150	0.243	22.416	< 0.001	3.158	1.962–5.082
Model 2	1.205	0.280	18.569	< 0.001	3.337	1.929–5.772
TyG index						
Unadjusted	2.348	0.379	38.484	< 0.001	10.467	4.985–21.981
Model 1	2.392	0.399	35.918	< 0.001	10.938	5.002–23.916
Model 2	2.587	0.463	31.150	< 0.001	13.287	5.357–32.956
HOMA-IR						
Unadjusted	0.146	0.051	8.351	< 0.001	1.157	1.048–1.278
Model 1	0.153	0.054	8.100	< 0.001	1.165	1.049–1.294
Model 2	0.153	0.058	6.955	< 0.001	1.166	1.040–1.307

Model 1: adjusted for basic characteristics including gender, age, family history of diabetes, presence of fatty liver, BMI, SBP, systolic blood pressure; DBP, diastolic blood pressure; Hg, hemoglobin; model 2: model 1 plus adjustments for high-density lipoprotein cholesterol (HDL-c), LDL-c, low-density lipoprotein cholesterol; ALT, alanine aminotransferase; AST, aspartate aminotransferase; γ-GGT, gamma-glutamyl transferase; ALP, alkaline phosphatase; UA, serum uric acid; BUN, blood urea nitrogen; FT3, serum creatinine, free triiodothyronine; FT4, free thyroxine and TSH, thyroid-stimulating hormone.

### Diagnostic value assessment of the TyG index

After establishing the independent associations between metabolic biomarkers and prediabetes, we evaluated their clinical utility as screening tools for this condition in children with obesity by conducting ROC curve analyses (**Table 3**). The 2hBG demonstrated the highest AUC of 0.950 (95% CI: 0.918–0.982, *p* < 0.001) with an optimal cutoff value of 7.760. At this threshold, the sensitivity for identifying PDM in children with obesity was 85.7%, and the specificity was 98.3%, indicating superior discriminative ability. The TyG index yielded an AUC of 0.776 (95% CI: 0.715–0.837, *p* < 0.001) at a cutoff of 8.895, corresponding to 55.1% sensitivity and 85.2% specificity. This performance surpassed that of TG (AUC = 0.714), FBG (AUC = 0.752), and HOMA-IR (AUC = 0.613), ranking second only to 2hBG.

**Table 3:** Diagnostic performance of individual and combined metabolic indicators for predicting prediabetes in children with obesity: results of ROC curve analysis.

Variable	AUC (95% CI)	Cutoff	Sensitivity	Specificity	Youden index	*P*-value
2hBG	0.950 (0.918–0.982)	7.760	0.857	0.983	0.840	< 0.001
FBG	0.752 (0.688–0.816)	5.705	0.469	0.887	0.356	< 0.001
HbA1c	0.757 (0.691–0.823)	5.550	0.653	0.809	0.462	< 0.001
TG	0.714 (0.644–0.783)	1.625	0.561	0.809	0.370	< 0.001
TyG index	0.776 (0.715–0.837)	8.895	0.551	0.852	0.403	< 0.001
HOMAIR	0.613 (0.538–0.689)	3.430	0.816	0.383	0.199	< 0.001

### Stratified analysis of TyG index tertiles: metabolic parameters and glycemic status in children with obesity

To investigate the potential association between the TyG index and PDM, 213 children and adolescents with obesity were stratified into three groups based on TyG index levels: T1 (≤ 8.42), T2 (8.43–8.89), and T3 (> 8.90). The comparative results of clinical and biochemical parameters among the groups are presented in **Table 4**.

**Table 4:** Clinical characteristics of children and adolescents with obesity by tertiles of TyG index.

Variable	Total (*N* = 213)	Tyg index tertiles			*P-*value
		T1 (≤ 8.42)	T2 (8.43–8.89)	T3 (> 8.90)	
Gender (boy/girl) (*n*)	145/68	48/23	49/22	48/23	0.979
Age at the time of visit (years)	12.86 ± 2.71	12.97 ± 2.78	13.21 ± 2.74	12.39 ± 2.59	0.177
Family history (*n*)	79 (37.1)	21 (29.6)	28 (39.4)	30 (42.3)	0.260
Fatty liver (%)	180 (84.5)	56 (78.9)	66 (93.0)	58 (81.7)	0.227
BMI (kg/m^2^)	30.12 ± 4.44	30.02 ± 4.62	31.20 ± 4.49	29.13 ± 3.99	0.020
BMI *Z*-score	2.91 ± 0.72	2.88 ± 0.75	3.02 ± 0.75	2.84 ± 0.64	0.285
SBP (mmHg)	120.88 ± 13.10	120.03 ± 11.72	121.82 ± 13.56	120.79 ± 14.04	0.718
DBP (mmHg)	79.10 ± 11.48	80.37 ± 12.05	78.83 ± 10.84	78.10 ± 11.57	0.488
Hg (g/L)	135.25 ± 13.14	134.11 ± 11.91	136.85 ± 13.36	134.80 ± 14.09	0.438
Lipids on admission					
TC (mmol/L)	4.16 (3.64, 4.66)	4.06 (3.31, 4.34)	4.11 (3.66, 4.66)	4.41 (3.98, 4.83)	0.001
TG (mmol/L)	1.35 (1.02, 1.98)	0.89 (0.74, 1.06)	1.35 (1.19, 1.55)	2.32 (1.97, 2.97)	< 0.001
HDL-c (mmol/L)	1.06 (0.94, 1.21)	1.14 (1.00, 1.35)	1.04 (0.94, 1.21)	1.03 (0.91, 1.13)	0.003
LDL-c (mmol/L)	2.48 (2.07, 2.89)	2.28 (1.79, 2.75)	2.50 (2.14, 2.83)	2.72 (2.27, 3.12)	0.003
Biochemical indicators					
ALT (U/L)	28.50 (19.60, 55.50)	26.00 (19.00, 40.90)	26.60 (18.30, 58.80)	35.40 (21.20, 82.10)	0.056
AST (U/L)	23.90 (18.45, 34.90)	22.20 (18.00, 28.70)	22.10 (16.70, 39.10)	27.00 (21.30, 51.00)	0.008
γ-GGT (U/L)	25.00 (17.90, 37.95)	23.00 (16.50, 31.00)	25.00 (18.00, 37.00)	30.00 (19.90, 47.60)	0.018
ALP (U/L)	199.00 (188.50, 270.50)	181.00 (97.00, 255.00)	187.00 (121.00, 263.40)	219.00 (144.00, 282.00)	0.124
Serum UA (µmol/L)	403.00 (344.00, 488.00)	383.30 (324.00, 467.00)	405.50 (355.00, 512.00)	419.00 (365.00, 506.00)	0.051
UN (mmol/L)	4.48 ± 1.11	4.48 ± 1.07	4.48 ± 1.20	4.49 ± 1.09	0.996
Serum creatinine (µmol/L)	45.00 (39.00, 55.00)	46.00 (42.00, 55.00)	46.00 (40.00, 57.00)	43.00 (37.00, 52.00)	0.037
Thyroid function					
FT3 (pmol/L)	6.23 ± 1.09	6.34 ± 1.18	6.12 ± 0.99	6.23 ± 1.10	0.466
FT4 (pmol/L)	16.22 ± 2.94	16.54 ± 3.22	16.33 ± 2.51	15.78 ± 3.04	0.288
TSH (µIU/mL)	2.95 (2.01, 4.08)	3.30 (2.03, 4.09)	2.81 (2.01, 3.76)	3.02 (1.84, 4.41)	0.582
Derived indicators					
HOMA-IR	4.54(3.27,6.47)	4.24(2.97,5.71)	4.33(3.28,7.14)	5.27(4.08,7.11)	0.005
TyG index	8.69 ± 0.56	8.11 ± 0.26	8.65 ± 0.13	9.29 ± 0.39	< 0.001
Hyperglycemia on admission					
FBG (mmol/L)	5.25 ± 0.77	4.86 ± 0.78	5.38 ± 0.70	5.50 ± 0.67	< 0.001
2hBG (mmol/L)	7.49 ± 1.39	6.97 ± 1.23	7.29 ± 1.30	8.20 ± 1.35	< 0.001
HbA1c (%)	5.46 ± 0.41	5.32 ± 0.34	5.39 ± 0.34	5.66 ± 0.45	< 0.001
FINS	20.22 (14.30, 28.27)	19.63 (12.40, 25.18)	19.88 (14.72, 28.37)	21.24 (16.03, 28.77)	0.136
Glucose metabolism state					
NGT	115 (54.0%)	57 (80.3%)	41 (57.7%)	17 (23.9%)	
IFG	12 (5.6%)	2 (2.8%)	5 (7.0%)	5 (7.0%)	
IGT	74 (34.7%)	11 (15.5%)	21 (29.6%)	42 (59.2%)	
IFG + IGT	12 (5.6%)	1 (1.4%)	4 (5.6%)	7 (9.9%)	
PDM	98 (46.0%)	14 (19.7%)	30 (42.3%)	54 (76.1%)	

Data are expressed as mean ± standard deviation, median (interquartile range), or *n* (%).

*IFG*, impaired fasting glucose; *IGT*, impaired glucose tolerance; *NGT*, normal glucose tolerance; *PDM*, prediabetes (includes IFG, IGT, and IFG + IGT).

With increasing TyG index levels, significant progressive elevations were observed in TG, LDL-c, AST, γ-GGT, HOMA-IR, FBG, 2hBG, and HbA1c (all *p* < 0.05). Conversely, HDL-c levels demonstrated a significant decline with a higher TyG index (*p* = 0.003). Regarding glucose metabolism status, the detection rate of PDM increased significantly across TyG index tertiles (19.7% in T1, 42.3% in T2, and 76.1% in T3). The proportion of participants with IGT was highest in the T3 group, while the percentage with NGT decreased from 80.3% in T1 to 23.9% in T3, showing a pronounced downward trend (*p* < 0.001).

### Graded increase in prediabetes odds by TyG index tertiles

As presented in **Table 5**, logistic regression analysis demonstrated a significant and robust positive association between the TyG index and PDM in children with obesity. Each 1-unit increment in the TyG index was associated with a markedly elevated risk of PDM, with adjusted OR of 10.467 (95% CI: 4.985–21.981), 10.938 (5.002–23.916), and 13.287 (5.357–32.956) across three multivariable-adjusted models. A dose-dependent gradient of increasing PDM risk was observed with ascending TyG index tertiles. Compared to T1 (≤ 8.42), participants in T2 (8.43–8.89) exhibited a 2.7–3.0-fold higher risk of PDM (OR range: 2.714–2.979), while those in T3 (> 8.90) showed a striking 12.9–13.1-fold elevated risk (OR range: 12.933–13.107).

**Table 5:** Dose-dependent association of TyG index with prediabetes risk in children with obesity.

Tyg index	Events	PDM OR (95% CI)		
		Model 1	Model 2	Model 3
Per 1 increment	98 (46.0%)	10.467 (4.985–21.981)	10.938 (5.002–23.916)	13.287 (5.357–32.956)
Tertiles				
T1 (≤ 8.42)	14 (19.72%)	1	1	1
T2 (8.43–8.89)	30 (42.25%)	2.979 (1.406–6.311)	2.837 (1.290–6.238)	2.714 (1.180–6.243)
T3 (> 8.90)	54 (76.06%)	12.933 (5.815–28.762)	13.107 (5.694–30.173)	12.974 (5.126–32.834)
*p* for trend		< 0.001	< 0.001	< 0.001

Data presented as odds ratio (95% confidence interval). *p* for trend calculated using TyG tertiles as ordinal variables.

Model 1: unadjusted; model 2: adjusted for basic characteristics including gender, age, family history of diabetes, presence of fatty liver, BMI, systolic blood pressure (SBP), diastolic blood pressure (DBP), and hemoglobin (Hg); model 3: model 2 plus adjustments for high-density lipoprotein cholesterol (HDL-c), low-density lipoprotein cholesterol (LDL-c), alanine aminotransferase (ALT), aspartate aminotransferase (AST), gamma-glutamyl transferase (γ-GGT), alkaline phosphatase (ALP), serum uric acid (UA), blood urea nitrogen (BUN), serum creatinine, free triiodothyronine (FT3), free thyroxine (FT4), and thyroid-stimulating hormone (TSH).

## Discussion

Our cross-sectional study systematically evaluated the association between the TyG index and PDM in 213 children and adolescents with obesity using LASSO regression-based variable selection, multivariate logistic regression, and ROC curve analysis. Our findings demonstrate that the TyG index exhibits a robust correlation with dysglycemia and serves as a promising screening tool for early PDM identification in children and adolescents with obesity.

Although the TyG index has been extensively validated in adult studies as a predictor of insulin resistance, T2DM, and cardiovascular diseases (33), evidence remains limited in pediatric populations. Our findings align with previous adult studies while expanding the evidence base for TyG index applications in pediatric populations, representing the first evaluation of its utility for PDM screening specifically in children and adolescents with obesity.

Key findings revealed that participants in the PDM group had significantly higher TyG index values than those in the NGT group. The TyG index showed positive correlations with traditional metabolic markers, including TG, LDL-C, liver enzymes, HOMA-IR, and HbA1c. Elevated TyG index levels were associated with increased prevalence of dyslipidemia, insulin resistance, and impaired glucose tolerance, consistent with Brito et al. (34), who concluded that the TyG index effectively predicts metabolic syndrome in adolescents and correlates strongly with hypertriglyceridemia and low HDL-c. Further supporting evidence (35) demonstrates high concordance between the TyG index and HOMA-IR in diagnosing insulin resistance among adolescents (kappa values: 0.902 for boys; 0.910 for girls), reinforcing the TyG index’s validity as a screening tool for pediatric insulin resistance. LASSO regression identified the TyG index, HOMA-IR, and HbA1c as the most PDM-relevant indicators. Subsequent logistic regression confirmed the TyG index as an independent risk factor for PDM, with a substantially higher OR (10.476, 95% CI: 4.985–21.981, *p* < 0.001) compared with isolated FBG (OR: 4.520, 95% CI: 2.727–7.493) or TG (OR: 3.104, 95% CI: 1.971–4.888). This suggests that synergistic amplification of glucose–lipid interactions enhances PDM risk detection. The TyG index outperformed HOMA-IR (OR: 1.157, 95% CI: 1.048–1.278) in PDM prediction, likely due to its ability to capture both lipid metabolism dysregulation and glucose homeostasis disruption. While HOMA-IR primarily reflects basal insulin resistance under fasting conditions, the TyG index combines fasting glucose and triglycerides, enabling the simultaneous detection of disturbances in glucose homeostasis and lipid metabolism. Elevated triglycerides suggest increased hepatic Very Low-Density Lipoprotein (VLDL) synthesis and impaired peripheral lipid clearance, whereas elevated blood glucose indicates insufficient insulin-mediated suppression of hepatic gluconeogenesis. The integration of these two parameters reflects hepatic insulin resistance, impaired skeletal muscle glucose utilization, and lipotoxicity-induced β-cell dysfunction. This comprehensive approach provides a more holistic view of the pathophysiological state driven by the interplay between glucose and lipid metabolism, which HOMA-IR fails to address (35, 36). This mechanistic advantage may explain the superior predictive performance of the TyG index for prediabetes, offering a strong theoretical basis for its application in pediatric clinical screening.

ROC analysis revealed significant discriminative power of the TyG index for PDM (AUC = 0.776, 95% CI: 0.715–0.837), comparable to HbA1c (AUC = 0.757, 95% CI: 0.691–0.823) and FBG (AUC = 0.752, 95% CI: 0.688–0.816). Stratification by optimal TyG index tertiles further confirmed a significant dose–response relationship with PDM risk (*p* < 0.001), demonstrating strong discriminatory power. Participants in the T3 exhibited nearly 13-fold increased PDM risk compared with those in T1. Regarding glucose metabolism status, the TyG index effectively distinguished children with IGT but showed no significant differentiation in individuals with IFG. Impaired fasting glucose and impaired glucose tolerance represent distinct metabolic abnormalities, with pathophysiological differences primarily involving hepatic versus peripheral insulin sensitivity. β-Cell dysfunction manifests differentially—individuals with IFG predominantly exhibit basal insulin secretion defects, whereas IGT is characterized by delayed postprandial insulin response. This dichotomy suggests that the TyG index may more sensitively reflect peripheral insulin resistance and postprandial β-cell secretory function. The underlying mechanism likely involves triglyceride–glucose metabolites interfering with skeletal muscle insulin signaling pathways and lipotoxicity-induced β-cell glucose desensitization. Elevated triglycerides typically indicate increased hepatic VLDL synthesis and impaired lipid clearance, while hyperglycemia reflects reduced insulin-mediated suppression of hepatic gluconeogenesis. These metabolic disturbances collectively contribute to systemic insulin resistance, a key mechanism in PDM pathogenesis (14, 37). Based on the mechanisms described above, enhancing the ability of the TyG index to identify IFG requires the integration of supplementary indicators that reflect hepatic IR and β-cell function. Therefore, future research could aim to develop multivariate prediction models by combining the TyG index with markers of hepatic steatosis (such as ALT) or β-cell function (such as HOMA-β). This approach is expected to achieve more accurate and comprehensive identification of different prediabetes phenotypes.

By integrating these two metabolic parameters, the TyG index serves as a comprehensive early marker of glucose metabolism disorders, particularly valuable in resource-limited settings where 2hBG measurement is unavailable. This mechanism, supported by He et al. (38) and Tahapary et al. (39) in adult populations, is now validated in pediatric populations through our study.

Compared to 2hBG testing, the TyG index requires only fasting glucose and triglyceride measurements, offering computational simplicity and cost-effectiveness that facilitate implementation in school-based screening, primary care, and large-scale pediatric health initiatives. Our findings confirm the robust and independent predictive value of the TyG index. Furthermore, we identified an optimal TyG cutoff value (8.90) and validated its discriminative capacity for high-risk subgroup classification. Park et al. (40) and Deng et al. (41) have noted cumulative metabolic risks associated with prolonged TyG index elevation, suggesting its potential utility in longitudinal monitoring.

Although the TyG index demonstrated a prominent performance in terms of the AUC, ranking second only to 2hBG, its sensitivity at the reported optimal cutoff value of 8.895 was only 55.1%, which is significantly lower than that of HbA1c and FPG. This suggests that in screening settings, the TyG index may fail to identify a portion of children with prediabetes, increasing the risk of false negatives and limiting its utility as a standalone screening tool.

However, the TyG index exhibited relatively high specificity (85.2%), indicating its advantage in reducing false positives and avoiding unnecessary further OGTT procedures. Clinically, the TyG index may be better suited as a stratified screening tool: in resource-limited primary healthcare or school-based health screenings, the TyG index could serve for preliminary identification of high-risk individuals, followed by HbA1c testing to improve sensitivity, thereby ensuring screening efficiency while minimizing the risk of missed diagnoses. Furthermore, future studies could explore the balance between sensitivity and specificity at different cutoff values or develop combined models incorporating other markers (such as HbA1c and HOMA-IR) to further optimize the application of the TyG index in pediatric prediabetes screening.

This study provides several novel contributions to the field of prediabetes screening in children and adolescents. First, to the best of our knowledge, it is among the few studies systematically evaluating the predictive value of the TyG index in a large sample of children and adolescents with obesity. Second, we identified the optimal cutoff value for the TyG index specific to this population, a threshold not previously reported in Chinese pediatric cohorts. Third, by directly comparing the TyG index with established markers such as HOMA-IR and HbA1c, we demonstrated that the TyG index exhibited comparable or even superior predictive performance in certain contexts. These findings enrich the existing knowledge base and further support the TyG index as a simple, cost-effective, and clinically feasible tool for the early identification of prediabetes in children and adolescents with obesity.

### Study strengths and limitations

Our study benefits from a relatively robust sample size (*n* = 213) with comprehensive metabolic profiling, employing multivariate adjustments and nonlinear modeling to enhance result reliability. The Chinese pediatric obesity data contribute to understanding the TyG index’s cross-ethnic applicability. However, several limitations must be acknowledged.

The cross-sectional design of this study precludes causal inferences regarding the relationship between the TyG index and prediabetes. Longitudinal studies are necessary to explore the temporal dynamics and causal effects of the TyG index in the progression of glucose metabolism abnormalities and the transition to diabetes. Additionally, single-center recruitment may introduce selection bias, and the homogeneous ethnic composition limits the generalizability of the findings. Variations in lipid metabolism, insulin sensitivity, and β-cell function across different ethnicities, as well as differences in age and pubertal stage, may influence the applicability of the TyG index.

Furthermore, discrepancies in prediabetes diagnostic criteria, healthcare systems, screening protocols, and laboratory conditions may impact the determination of the TyG index’s cutoff values and predictive performance. The study also did not account for unmeasured confounders, including dietary patterns, physical activity, and psychological factors, which may lead to an overestimation of the true strength of this association. However, after adjusting for several strong confounders closely related to lifestyle, such as age, gender, and particularly BMI, in the multivariate logistic regression model, the TyG index still showed a strong and independent association with prediabetes (OR = 13.287), suggesting that its effect is unlikely to be entirely driven by unmeasured lifestyle factors. Integrating multiomics data in future studies could help refine predictive modeling, improving the accuracy of the TyG index.

### Future directions

Future research should focus on several key areas to address the study’s limitations and expand the clinical applicability of the TyG index. First, longitudinal cohort studies are needed to validate the causal role and temporal effects of the TyG index in predicting the progression of glucose metabolism abnormalities and the transition to diabetes. Second, large-scale, multicenter, multiethnic studies in diverse healthcare settings should be conducted to assess the stability of the TyG index cutoff values and to explore its applicability across different populations.

Additionally, combining the TyG index with other metabolic markers, such as HbA1c, BMI, family history, or inflammatory markers, could help construct more efficient predictive models. Feasibility studies in school-based or primary healthcare settings will also be essential for facilitating the clinical translation of the TyG index in early prediabetes screening in children and adolescents.

## Conclusions

The TyG index demonstrates a strong association with PDM risk and excellent independent predictive capacity in children and adolescents with obesity. As a simple, economical tool requiring no insulin measurement, it shows promise for early PDM identification and risk stratification in pediatric populations. Future multicenter, longitudinal cohort studies should further validate its predictive value for diabetes progression and clinical utility.

## Ethics approval and consent to participate

This study was conducted in accordance with the Declaration of Helsinki. Ethical approval was granted by the Ethics Committee of Henan Provincial People’s Hospital (Approval No. [2023]; Ethics No. [49]). Written informed consent was obtained from the parents or legal guardians of all participants. For minors capable of providing informed assent, both parental consent and the minors’ assent were obtained in accordance with ethical guidelines. Institutional permission was also granted by the hospital administration.

## Data Availability

The raw data supporting the conclusions of this article will be made available by the authors, without undue reservation.
